# Comparative Genomics Reveals Evidence of Genome Reduction and High Extracellular Protein Degradation Potential in *Kangiella*

**DOI:** 10.3389/fmicb.2018.01224

**Published:** 2018-06-07

**Authors:** Jiahua Wang, Ye Lu, Muhammad Z. Nawaz, Jun Xu

**Affiliations:** ^1^Institute of Oceanography, Shanghai Jiao Tong University, Shanghai, China; ^2^State Key Laboratory of Microbial Metabolism, School of Life Sciences and Biotechnology, Shanghai Jiao Tong University, Shanghai, China

**Keywords:** marine bacteria, Oceanospirillales, *Kangiella*, genome reduction, protein degradation

## Abstract

The genus *Kangiella* has recently been proposed within the family Kangiellaceae, belonging to order Oceanospirillales. Here, we report the complete genome sequence of a novel strain, *Kangiella profundi* FT102, which is the only *Kangiella* species isolated from a deep sea sediment sample. Furthermore, gaps in the publicly available genome scaffold of *K. aquimarina* DSM 16071 (NCBI Reference Sequence: NZ_ARFE00000000.1) were also filled using polymerase chain reaction (PCR) and Sanger sequencing. A comparative genomic analysis of five *Kangiella* and 18 non-*Kangiella* strains revealed insights into their metabolic potential. It was shown that low genomic redundancy and *Kangiella*-lineage-specific gene loss are the key reasons behind the genome reduction in *Kangiella* compared to that in any other free-living Oceanospirillales strain. The occurrence of relatively diverse and more frequent extracellular protease-coding genes along with the incomplete carbohydrate metabolic pathways in the genome suggests that *Kangiella* has high extracellular protein degradation potential. Growth of *Kangiella* strains has been observed using amino acids as the only carbon and nitrogen source and tends to increase with additional tryptone. Here, we propose that extracellular protein degradation and amino acid utilization are significant and prominent features of *Kangiella*. Our study provides more insight into the genomic traits and proteolytic metabolic capabilities of *Kangiella*.

## Introduction

The ocean covers 71% of the earth’s surface and is regarded as the largest habitat for life on the planet Earth. Marine microorganisms are known to play an essential role in energy conservation and biogeochemical cycling in the oceans. Heterotrophic prokaryotes are considered key players in the decomposition of the dissolved organic matter (DOM) and particulate organic matter (POM) present therein (DeLong and Karl, 2005; Azam and Malfatti, 2007).

Oceanospirillales is an order of proteobacteria with seven families of heterotrophic marine bacteria that are usually associated with oil spills and are known to be involved in xylan and hydrocarbon utilization (Choi et al., 2012; Cao et al., 2014). Recently, a new family named Kangiellaceae has been proposed within the order Oceanospirillales based on phylogenetic, chemotaxonomic and physiological characteristics; this family comprises three genera: *Kangiella, Aliikangiella*, and *Pleionea* (Wang et al., 2015). Although the *Kangiella* genus was reclassified within Kangiellaceae instead of Alcanivoracaceae based on 16S rRNA gene phylogeny (Wang et al., 2015), taxonomic signatures at the genomic level are still needed.

*Kangiella* are gram-negative long rods that are non-motile and non-spore-forming bacteria. Nine strains belonging to the genus *Kangiella* have been isolated from various marine environments, including marine sand (Kim et al., 2015), tidal flat sediments and coastal regions (Yoon et al., 2004, 2012; Romanenko et al., 2010; Ahn et al., 2011; Jean et al., 2012; Lee et al., 2013). For example, both *K. koreensis* DSM 16069 and *Kangiella aquimarina* DSM 16071 were isolated from tidal flat sediment at Daepo Beach, Yellow Sea, Korea, and were shown to grow optimally at 30–37°C and pH 7.0–8.0 (Yoon et al., 2004). *K. sediminilitoris* KCTC 23892 was isolated from tidal flat sediment from the South Sea in South Korea, which was shown to grow optimally at 30–37°C and pH 7.0–7.5 (Lee et al., 2013). *Kangiella geojedonensis* KCTC 23420 was isolated from seawater off the southern coast of Korea, and was shown to grow optimally at 10–40°C and pH 7.0–7.5 (Yoon et al., 2004). Recently, we isolated a novel strain named *Kangiella profundi* from a deep sea sediment sample that was collected from the southwest Indian Ocean at a depth of 2784 m (Xu et al., 2015). Due to limited research, our understanding about the metabolic potential and ecological functions of bacteria belonging to the family Kangiellaceae is still obscure.

To date, no genome has been completely sequenced from either of the two genera *Aliikangiella* and *Pleionea*. Moreover, only three completely sequenced genomes of the genus *Kangiella*, i.e., *K. koreensis* DSM 16069 (GenBank accession: GCA_000024085.1), *K. sediminilitoris* KCTC 23892 (GenBank accession: GCA_001708405.1), and *K. geojedonensis* KCTC 23420 (GenBank accession: GCA_000981765.1), are available. In this study, we report the complete genome sequence of *K. profundi* FT102 (GenBank accession: CP025120). Comparative genomics-based approaches were utilized to explore the metabolic potential of *Kangiella*. We show that featured genomic reduction in *Kangiella* is due to low genomic redundancy as well as *Kangiella* lineage-specific gene loss. The metabolic base of *Kangiella* as a powerful extracellular protein degrader was investigated. Our study provides the first insights into the genomic and metabolic capabilities of *Kangiella*.

## Materials and Methods

### Bacterial Strains and Growth Conditions

*Kangiella profundi* FT102*^T^* ( = CGMCC 1.12959*^T^* = KCTC 42297*^T^* = JCM 30232*^T^*) is from our laboratory stock. The strains *K. koreensis* DSM 16069 (JCM 12317), *K. aquimarina* DSM 16071 (JCM 12318) and *K. geojedonensis* KCTC 23420 were obtained from Japan Collection of Microorganisms (JCM) and Korean Collection for Type Cultures (KCTC). These *Kangiella* strains were grown in marine broth 2216 (Xu et al., 2015) and marine peptone medium (1 L of broth containing 1 g of yeast extract, 10 g of tryptone, and 1000 mL of artificial sea water; pH adjusted to 7.3) at 37°C and 200 rpm under aerobic conditions. Bacterial growth was measured by an automatic turbiditimetry using a Bioscreen C analyzing system (Labsystems).

### Cultivation in Defined Medium

The defined cultivation method for *Kangiella* has been described elsewhere (Widdel et al., 2006). All 20 common amino acids, as a sole source of carbon, nitrogen and energy, were filter-sterilized and added to the media at a final concentration of 0.2 g/L of each amino acid. The medium contained (g/L): NaCl (26.0), MgCl_2_⋅6H_2_O (5.0), CaCl_2_⋅2H_2_O (1.4), NH_4_Cl (0.3), KH_2_PO_4_ (0.1), KCl (0.50), NaNO_3_ (1.0), with (mg/L), EDTA, disodium salt (5.20), FeSO_4_⋅7H_2_O (2.10), and (μg/L): H_3_BO_3_ (10.0), MnCl_2_⋅4H_2_O (5.0), CoCl_2_⋅6H_2_O (190.0), ZnSO_4_⋅7H_2_O (144.0), CuCl_2_⋅2H_2_O (10.0), Na_2_MoO_4_⋅2H_2_O (36.0), NiCl_2_⋅6H_2_O (24.0). Vitamins were filter-sterilized and added to final concentrations (μg/L) of 4-Aminobenzoic acid (4.0), D(+)-biotin (1.0), Nicotinic acid (10.0), D(+)-Pantothenic acid, calcium salt (5.0), Pyridoxine-HCl (15.0), Cyanocobalamin (5.0), and Thiamine-HCl (10.0).

### Genomic DNA Extraction

Genomic DNA was extracted as described by Chen et al. (2005). The quality and quantity of the extracted genomic DNA were checked by agarose gel electrophoresis and a NanoDrop spectrophotometer (Thermo Scientific).

### Genome Sequencing and Assembly

Genomic DNA of *K. profundi* FT102 was sequenced using a next-generation sequencing platform. Illumina HiSeq 2000 (2^∗^120bp, 0.5-kb insert size) sequencing was performed by BGI (Shenzhen, China), and *de novo* assembly was performed by using the Velvet assembler v.1.2.10 (Zerbino and Birney, 2008) with the following parameters: min kmer 31; max kmer 99; kmer step 6; and insert length 500. Eight contigs were assembled, including one containing an rRNA operon with doubled coverage and two short ones (< 550 bp). Then, these contigs were ordered according to the completed genome of *K. koreensis* DSM 16069. All gaps were filled using polymerase chain reaction (PCR) and Sanger sequencing (**Supplementary Material S1**). The coding sequence of *dnaA* was set as the genomic start in the positive strand.

### Genomic Data Collection

We collected five *Kangiella* (including *K. profundi* FT102) and 18 genomes of other Oceanospirillales (**Table 1**) from the NCBI database^1^. Both nucleotide and protein sequences were used in this study. The scaffold genome of *K. aquimarina* DSM 16071 was completed by PCR and sequencing in the present study.

**Table 1:** Genomic features of the *Kangiella* strains used in present study.

Species name	*Kangiella*	*Kangiella*	*Kangiella*	*Kangiella*	*Kangiella*
	*aquimarina*	*geojedonen*	*koreensis*	*profundi*	*sediminilitoris*
	DSM 16071	*sis* KCTC 23420	DSM 16069	FT102	KCTC 23892
Genome Size	2,686,123	2,495,242	2,852,073	2,653,010	2,496,140
%GC	43.72%	43.78%	43.69%	43.81%	43.40%
tRNA operon	41	41	41	41	41
rRNA number	2	2	2	2	2
ORF number	2446	2208	2632	2484	2308
ORF total length	2,438,865	2,244,783	2,594,526	2,382,009	2,250,390
%ORF length	90.79%	89.96%	90.97%	89.79%	90.15%
SignalP	317	298	339	305	283
%signalP	12.96%	13.50%	12.88%	12.28%	12.26%
TM	606	533	701	610	581
%TM	24.78%	24.14%	26.63%	24.56%	25.17%
No. of Paralogs	130	121	150	142	133
ORF in paralogs	344	296	396	370	332


*SingnalP, means signal peptide-fused protein; %SignalP, means percentage of signal peptide-fused proteins; TM, means transmembrane protein; %TM, means percentage of transmembrane proteins.*

### Genome Annotation and Comparative Analysis

Replication origins (*oriC*s) in bacterial genomes were predicted using Ori-Finder^2^, which predicts based on analysis of the base composition asymmetry using the Z-curve method, distribution of DnaA boxes, and the occurrence of genes frequently close to *oriC*s (Gao and Zhang, 2008). Putative protein-coding sequences were predicted using Glimmer 3 (Delcher et al., 1999), which was trained with the CDS (coding sequences) from four completely sequenced *Kangiella* genomes. Manual curation of all coding sequences was performed by examining the database hits of BLAST 2.2.25+against the NR database and the RAST server (Aziz et al., 2008). tRNA and rRNA genes were predicted using tRNAscan-SE 1.3.1 (Lowe and Eddy, 1997) and RNAmmer 1.2 (Lagesen et al., 2007), respectively. Signal peptides of the ORFs were predicted with SignalP 4.1 (Petersen et al., 2011; **Supplementary Material S3**). Peptidase genes were predicted with MEROPS batch BLAST (Rawlings et al., 2016; **Supplementary Material S3**).

Protein families of 23 Oceanospirillales strains including 5 *Kangiella* were clustered using a local OrthoMCL 2.0.9 (Li et al., 2003) with the following cut-off values: identity, 30%; coverage, 50%; *E*-value, 1e-5; score, 40; and MCL Markov clustering inflation index, 1.5. To identify the *Kangiella* signature proteins, the core genome components were blasted using BLASTP against the non-redundant protein sequence (nr) database, excluding the *Kangiella* genus. A protein was considered a *Kangiella* signature protein if there were no BLAST hits with acceptable *E*-values (<10^-1^), similarity (>20%), or coverage (>50%). GO (gene ontology) term enrichment was performed using Blast2GO PRO 3.0. COG (cluster of orthologous genes) classification was performed using the WebMGA online server^3^.

### Metabolic Network of *Kangiella*

The core metabolic network of *Kangiella* was constructed based on the core genome information of the five *Kangiella* strains using KEGG BlastKOALA^4^.

### Genome Rearrangement Analysis

Locally collinear blocks (LCBs) of the five *Kangiella* strains were investigated using Mauve aligner 2.4.0 (Darling et al., 2004). Each LCB represents a region of homologous sequence without rearrangement among genomes.

### GC-Skew Analysis

GenSkew online application^5^ was used to compute and plot GC-skew data.

## Results

### Genomic Features of *Kangiella profundi* FT102

**FIGURE 1:**
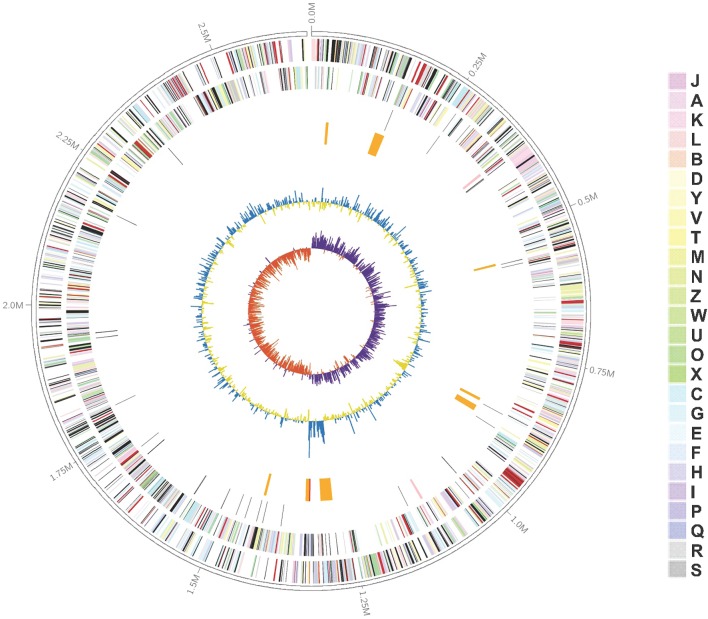
Graphical representation of the *Kangiella profundi* FT102 genome. Genes on the forward (shown in **Outer** circle) and reverse (shown in **Inner** circle) strands are colored according to their cluster of orthologous genes (COG) categories, RNA genes are highlighted with different colors (tRNAs orange, rRNAs red, other RNAs green), gene islands and GC content are shown in black, and GC skew is shown in light green/orange.

The complete genome sequence of *K. profundi* FT102 is composed of a circular chromosome of 2,653,010 bp with 43.81% GC content (**Figure 1**). The coding region covers 89.79% of the genome, encoding 2,484 proteins, of which 2,042 proteins were annotated with COG classification (**Supplementary Table S1**). Translation, ribosomal structure, general function prediction only (R), biogenesis (J), and amino acid transport, and metabolism (E) were the most abundant COG categories (7.81, 6.60, and 6.42%, respectively). The genome also encodes 41 tRNAs, as well as 2 rRNA operons. Signal peptides and transmembrane helices account for 12.28% (305) and 24.56% (610) of the protein-coding genes, respectively (**Table 1**).

Moreover, eight genomic islands (GIs) were predicted in the *K*. *profundi* FT102 strain (**Figure 1**). One of the GIs encodes 15 proteins (orf01390 to orf01530), including a putative type VI secretion system and a related lysozyme, which might provide antibacterial ability to *K*. *profundi* FT102. Another GI with the least GC content compared to the rest of the genome was predicted to harbor gene clusters for the biosynthesis of capsular polysaccharides. In contrast, the region with the highest GC content, located next to the GC-skew, was annotated as a putative heavy-metal resistance island against Zn/Co/Cd.

### Signature Proteins of the Genus *Kangiella*

The *Kangiella* genus is of phylogenetic interest because of its very isolated location in Oceanospirillales. *K. koreensis* is the first type species of this genus to be described and was selected for whole-genome sequencing as part of the Genomic Encyclopedia of Bacteria and Archaea project (Han et al., 2009). With five complete genomes at hand, we identified the signature proteins of *Kangiella* by BLAST analysis on the core genome with the following cutoff: at least 60% identity (*e*-value: 1e-30) with each other in the same orthologous families and less than 30% identity (*e*-value: 1e-1) with any non-*Kangiella* hits from the NR database. Under the cutoff, four orthologous families (KangOF24, 566, 656, and 804) appeared as *Kangiella* specific and were annotated as hypothetical proteins. Notably, two copies of KangOF24 were observed in *K. sediminilitoris* KCTC 23892 (AOE50183.1 and AOE50181.1). These signature proteins could be used as genus-specific molecular markers to identify *Kangiella* independently of detection of 16S rRNA gene as a phylogenic marker.

### Analysis of Orthologous Gene Families

**FIGURE 2:**
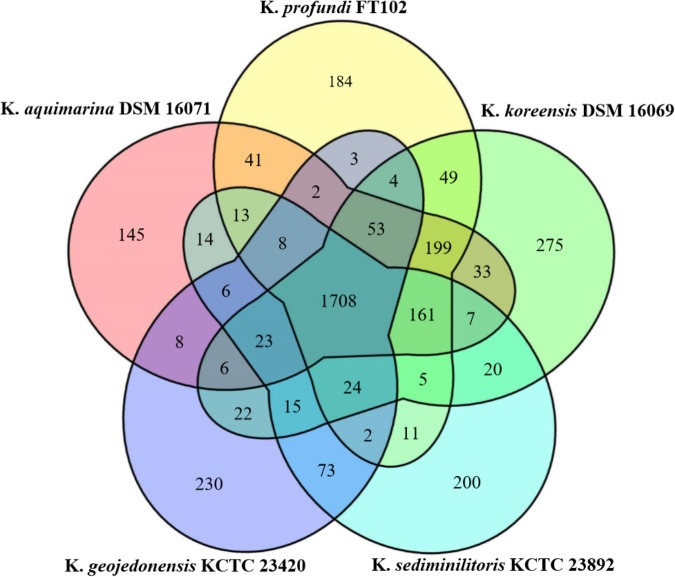
Venn diagram of the orthologous families of the five *Kangiella* species. The size of the core genome is shown in the center, while the numbers of strain-specific genes in *Kangiella* strains are shown in the petals.

The genomic features of the five *Kangiella* species used in the present study are shown in **Table 1**. The Markov clustering algorithm OrthoMCL was used to identify orthologous gene families. Further analysis of these families in the five *Kangiella* genomes revealed 3,544 orthologs (**Figure 2** and **Supplementary Table S2**), which includes (1) 1,708 core genome components, of which only 21 orthologs contain duplication in at least one genome (**Supplementary Table S3**); (2) 802 components in the dispensable genome, which have representatives in at least two but not all of the five genomes; and (3) 1,034 components that were uniquely present only in one genome, which include only 10 lineage-unique (LSE) gene families and 1,024 singletons. According to the COG classification (**Supplementary Figure S1** and **Supplementary Table S4**), it is evident that core genes mainly belong to categories J (translation, ribosome structure, and biogenesis), C (energy production and conversion), D (cell cycle control, cell division, and chromosome partitioning), F (nucleotide transport and metabolism), G (carbohydrate transport and metabolism), H (coenzyme transport and metabolism), I (lipid transport and metabolism), and L (replication, recombination, and repair); the unique genes are involved in V (defense mechanisms); and dispensable genes are found more often associated with P (inorganic ion transport and metabolism), and T (signal transduction mechanisms).

### Strain-Specific Losses and Gains in the Metabolic Pathways in *Kangiella*

To determine the differences in the metabolic potential among *Kangiella* strains, we compared the GO terms from each strain, and strain-specific gene gains and losses were identified. To avoid the biases of annotation, “gained” GO terms were derived from strain-specific orthologous families that were not possessed by any of the other four strains. On the other hand, “lost” GO terms were retrieved only from those dispensable orthologous families that were owned by each of the four strains and missing in only one. Genes related to these GO terms were manually checked using both KEGG pathways and BLASTP analysis.

The results suggest that *K. profundi* FT102 gained a type III restriction endonuclease (orf15860) and a related DNA methyltransferase (orf15870), as well as a type VI secretion system. The gene clusters related to sulfide reduction and arginine biosynthesis were missing in the FT102 strain; this absence was also validated through PCR and sequencing (**Supplementary Figure S2** and **Supplementary Material S2**).

*Kangiella aquimarina* DSM 16071 was found to be the only strain with a type I restriction-modification system, including site-specific deoxyribonuclease activity (WP_033414025.1) and related methyltransferase (WP_033414024.1). Moreover, 2-isopropylmalate synthases were observed in other analyzed strains but were absent in *K. aquimarina* DSM 16071. 2-isopropylmalate is an important substrate for the biosynthesis of leucine from pyruvate and could be catalyzed to leucine along with acetyl-CoA, 3-methyl-2-oxobutanoate, and water. However, *K*. *aquimarina* DSM 16071 contains isopropylmalate isomerase (WP_018624573.1), which could transform (2R,3S)-3-isopropylmalate to (2S)-2-isopropylmalate. Therefore, it was suggested that *K*. *aquimarina* DSM 16071 could synthesize leucine, similar to other species, using an alternate pathway.

*Kangiella koreensis* DSM 16069 represents the largest genome size compared to the other four strains used in this study, and no strain-specific GO term loss was found in *K. koreensis* DSM 16069. Furthermore, *K. koreensis* DSM 16069 was found to exhibit putative nitrate reduction capability. A gene cluster with at least 19 proteins (ACV26744.1-ACV26762.1) and 12 GO terms related to Mo-molybdopterin cofactor biosynthesis and dissimilatory nitrate reduction were found limited to *K*. *koreensis* DSM 16069.

Although, the genome size of *K. geojedonensis* KCTC 23420 is the smallest among all the strains used herein, it was the only one predicted to have a putative capacity for synthesizing all 20 amino acids. A gene cluster (AKE51219.1–AKE51221.1) related to branched-chain amino acid biosynthetic and metabolic processes was found in *K. geojedonensis* KCTC 23420, while it was missing in the rest of the four *Kangiella* strains. Furthermore, the presence of homoserine kinase (AKE52760.1) possibly represents a strain-specific biosynthetic pathway from L-aspartate to threonine via O-phospho-L-serine in *K. geojedonensis* KCTC 23420. Additionally, 10 strain-specific GO terms in 6 orthologous families were found in the second island of *K. geojedonensis* KCTC 23420 (consisting of 21 protein-coding genes) and are involved in capsule biosynthesis. However, they lack genes involved in bacterial respiration (such as cytochrome c oxidase subunits and HemN), oxidative resistance (such as superoxide dismutase), heavy metal transport, and nitrite utilization.

*Kangiella sediminilitoris* KCTC 23892 was the only strain with an ammonium transmembrane transporter (AOE50237.1), while it was also the only one among the five *Kangiella* strains that lacked aspartate-ammonia ligase, whose function could be supplemented by asparagine synthase (AOE49243.1).

### Reconstruction of the Basic Metabolic Pathways of Carbohydrate and Amino Acid

**FIGURE 3:**
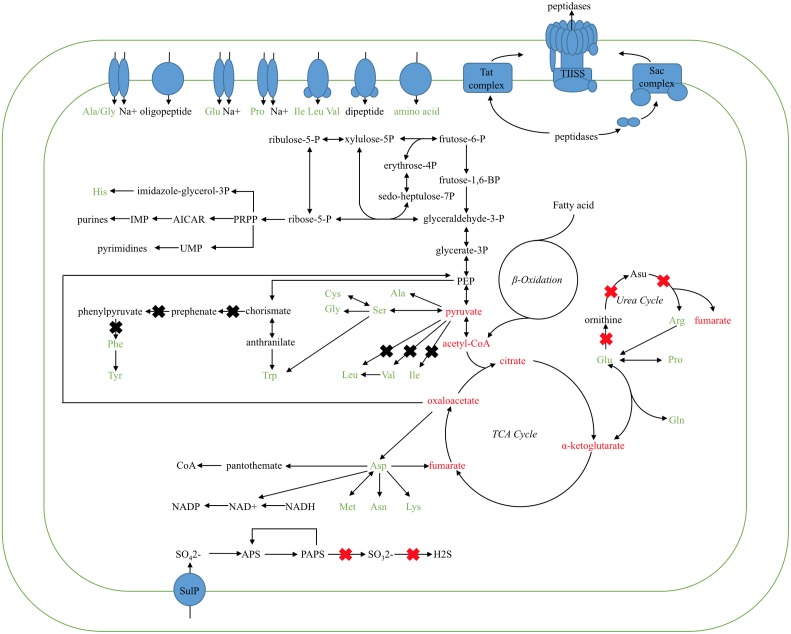
Proposed metabolic pathways of the TCA cycle, inferred from the *Kangiella* core genome. Ovals in blue represent the amino acid/oligopeptide transporters and protein-secreting systems. Black arrows show the flow of the metabolic pathway. X marks in the red color show the genes absent only in *K. profundi* FT102 (confirmed by PCR and growth experiments), whereas X marks in black represent the genes absent in *K. profundi* FT102 and at least one other species (but not all the species used here).

Based on the comparison KEGG pathway analysis described above, the main metabolic network of the *Kangiella* strains were reconstructed. The glycolysis, tricarboxylic acid (TCA) cycle, and amino acid biosynthesis and transport pathways are shown in **Figure 3**. Although *Aliikangiella marina* GYP-15*^T^* was reported as a carbohydrate utilizer (Wang et al., 2015), the gene encoding carbohydrate transporter or glucose kinase was not predicted in the *Kangiella* strains, which might be the reason that the heterotrophic growth of *Kangiella* strains could not be supported when using glucose as the sole carbon source (data not shown). Therefore, it is possible that carbohydrates are derived from central carbon metabolism (such as pyruvate) and transformed via reverse glycolysis and the pentose phosphate pathway. Fatty acids could be one of the carbon sources, as the genes coding for the key enzymes in the fatty acid beta-oxidation pathway could be found in the genomes of *Kangiella*. Amino acids could also feed central carbon metabolism via deamination and/or transamination, and most of them could be synthesized by each strain.

**FIGURE 4:**
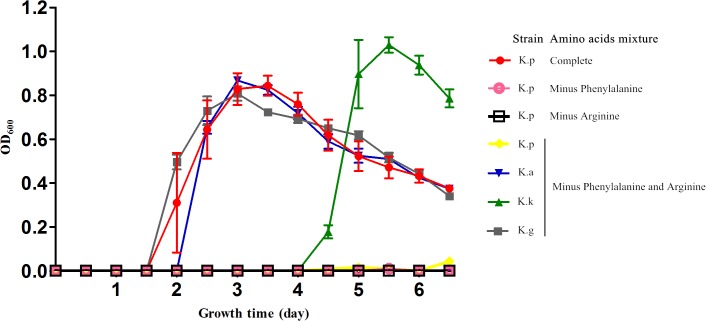
Growth curve of the four *Kangiella* strains cultured in the defined amino acids medium. K.p, *Kangiella aquimarina* DSM 16071; K.k, *Kangiella koreensis* DSM 16069; K.g, *Kangiella geojedonensis* KCTC 23420; and K.p, *Kangiella profundi* FT102. Complete: 20 amino acids; Minus Phenylalanine: phenylalanine-absent 19 amino acids; Minus Arginine: arginine-absent 19 amino acids; Minus Phenylalanine and Arginine: phenylalanine- and arginine-absent 18 amino acids.

Diversity in metabolic capability in the biosynthesis of certain amino acids, including valine, isoleucine, arginine, and phenylalanine, was observed among the analyzed strains (**Figure 3**). *K. geojedonensis* KCTC 23420 appeared to be able to synthesize valine and isoleucine, while *K. profundi* FT102 could not synthesize arginine. Additionally, biosynthesis of phenylalanine was found to be missing in both *K. profundi* FT102 and *K. sediminilitoris* KCTC 23892 because of the loss of a gene cluster containing phospho-2-dehydro-3-deoxyheptonate aldolase and prephenate dehydratase (**Supplementary Figure S2**). Particularly, the predicted disability of the phenylalanine and arginine biosynthesis in *K. profundi* FT102, were experimentally validated by growth curve analysis under various defined media with amino acids as the sole carbon and nitrogen source (**Figure 4**). Interestingly, we detected various kinds of secretory peptidases, which are exported by TIISS/Tat/Sac system to degrade extracellular proteins into amino acids and peptides in these strains. Furthermore, seven types of amino acid/peptide transporters were also identified, which could support the intake of these amino acids/peptides from the extracellular environment.

### Genome Reduction and Metabolic Simplicity of *Kangiella* Strains

**FIGURE 5:**
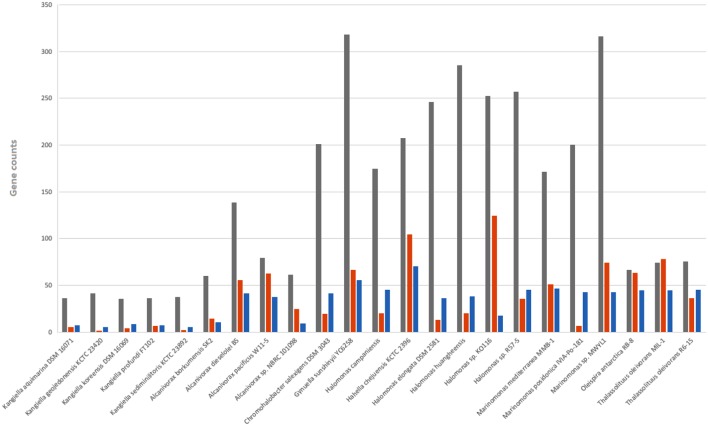
Number of genes belonging to selected COG categories in all 23 strains in Oceanospirillales. G shown in gray is related to carbohydrate transport and metabolism; N shown in orange is related to cell motility; and X shown in blue is related to mobility.

The genomic size of the *Kangiella* strains is smaller than that of any other free-living Oceanospirillales strains. To gain insights into the genome reduction in *Kangiella*, we performed comparative genome analysis with 18 non-*Kangiella* completely sequenced genomes of Oceanospirillales strains (**Supplementary Table S5**). We particularly focused on the investigating genomic redundancy, functional gene categories and genome function reduction. OrthoMCL clustering results showed that each *Kangiella* strain possesses at most 150 paralogous gene families within 396 genes, whereas non-*Kangiella* strains exhibit at least 351 paralogous families within 1,118 genes, demonstrating that low genomic redundancy is the primary reason behind the genome reduction. There were fewer COG categories in *Kangiella* strains than in their non-*Kangiella* counterparts (**Supplementary Table S1**), especially for carbohydrate transport and metabolism (COG category G), genes in the mobilome (COG category X) and cell motility (COG category N). Each *Kangiella* strain exhibits no more than 41 COG category G orthologous proteins, while non-*Kangiella* strains have at least 60 COG category G, demonstrating that *Kangiella* has a decreased preference for carbohydrate transport and metabolism. On the average, *Kangiella* strains have only four COG category X orthologous proteins, while non-*Kangiella* strains have 48, which reflects less horizontal gene transfer during the evolution of *Kangiella*. There also seemed to be fewer COG category N orthologous proteins than the average in non-*Kangiella* strains, which might be mainly because *Kangiella* strains lack flagellum (**Figure 5**).

To gain insights into the reduction in *Kangiella*-specific function, we clustered all five *Kangiella* protein sequences together with their 18 counterparts using OrthoMCL. Among 23,058 Oceanospirillales orthologous families (OceaOFs) (**Supplementary Table S6**), we found 96 families that were only absent in *Kangiella* and called them “*Kangiella*-specific-absences.” Genes both from these families and all five *Kangiella* genomes were annotated against the GO database. If a GO term from these “*Kangiella*-specific-absent” families was also found to be absent in all the *Kangiella* genomes, this pathway was inferred to be lost in *Kangiella*. Approximately 31 GO terms from 24 OceaOFs were found missing in *Kangiella* only (**Supplementary Table S7**). All the non-*Kangiella* Oceanospirillales were found to contain a phosphoenolpyruvate-dependent sugar phosphotransferase system; in contrast, this protein was missing in *Kangiella*. Glycerol is the primary carbon source available to halophilic heterotrophic communities. Glycerol kinase (EC 2.7.1.30; ATP-glycerol 3-phosphotransferase) is required for glycerol metabolism, and *Kangiella* strains were found to be deprived of it, suggesting that glycerol is not the carbon source in *Kangiella*. Malate synthase works together with isocitrate lyase in the glyoxylate cycle to bypass two oxidative steps of the Krebs cycle and permit carbon incorporation from acetate or fatty acids in many microorganisms. *Kangiella* is also lacking these enzymes, which demonstrates the inertness of gluconeogenesis in *Kangiella*. Phosphoenolpyruvate carboxylase was also missing in *Kangiella*. In the aspect of amino acid biosynthesis and metabolism, the glutamate synthase, L-alanine:2-oxoglutarate aminotransferase and threonine dehydratase are missing in all *Kangiella* genomes. *Kangiella* strains also lack GO:0008942 (F:nitrite reductase [NAD(P)H] activity) and GO:0042128 (P:nitrate assimilation), which suggests that *Kangiella* cannot perform assimilatory nitrate reduction. Many halophilic microorganisms accumulate ectoine (1,4,5,6-tetrahydro-2-methyl-4-pyrimidine carboxylic acid) to counteract heat, cold, desiccation, and high salinity. The operon with L-2,4-diaminobutyric acid acetyltransferase, 4-aminobutyrate aminotransferase, and ectoine synthase is missing in *Kangiella*.

### High Extracellular-Protein/Peptide Degradation Potential of *Kangiella*

**FIGURE 6:**
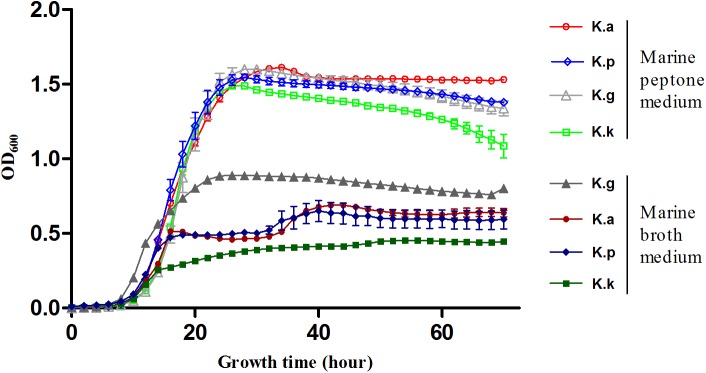
Growth curve of four *Kangiella* strains cultured in marine peptone medium grouped in “Marine peptone medium” and marine broth 2216 grouped in “Marine broth medium.” K.a, *K. aquimarina* DSM 16071; K.k, *K. koreensis* DSM 16069; K.g, *K. geojedonensis* KCTC 23420; and K.p, *K. profundi* FT102.

The low genomic redundancy in *Kangiella*, was observed in the present study (**Supplementary Table S3**). Surprisingly, only 21 (1.23%) orthologs with duplications in the core genome and five of them (KangOF4, 8, 9, 15, and 19) were annotated to be SignalP-fused peptidases (SignalP) that involved in proteolysis (GO:0006508). Our experimental results also demonstrated that *Kangiella* strains could grow solely on amino acids as the carbon and nitrogen source (**Figure 4**). In addition, the biomass of *Kangiella* was increased if tryptone in marine broth 2216 medium has been doubled to make a enriched marine peptone medium (**Figure 6**). Therefore, we proposed that extracellular protein degradation and amino acid utilization are essential and prominent features of *Kangiella*.

Although the genome sizes of *Kangiella* strains were smaller than those of the other genus in Oceanospirillales, the percentage of SignalP-fused peptidases in *Kangiella* strains (1.68–2.36%) was significantly higher than that in the other genomes belonging to the order Oceanospirillales (0.25–0.80%) (**Table 2** and **Supplementary Table S8**). Furthermore, *Kangiella* strains also exhibit diverse types of peptidases. Approximately 67–71 peptidase subfamilies were predicted in *Kangiella*, of which 26 subfamilies were signal-fused peptidases, whereas only 55 subfamilies were identified in their non-*Kangiella* counterparts on average, and only 14 were signal-fused peptidases. Moreover, eight families of peptidases with SignalP (S8A, S9B, S9C, S41A, M19, M16B, and M38) were found to be remarkably abundant in *Kangiella*. We also identified three *Kangiella*-specific SignalP-fused protease families (S10, S46, and M28D).

**Table 2:** Comparison of peptidases in all the 23 genomes used in this study.

Sr. No.	Strain name	Total No. of	Secretory	No. of Secretory
		Peptidase	peptidase	Peptidase/MB
1	*Kangiella aquimarina* DSM 16071	142	56	20.85^∗^
2	*Kangiella geojedonensis* KCTC 23420	140	50	20.04^∗^
3	*Kangiella koreensis* DSM 16069	156	62	21.74^∗^
4	*Kangiella profundi* FT102	142	54	20.40^∗^
5	*Kangiella sediminilitoris* KCTC 23892	142	43	17.23^∗^
6	*Alcanivorax borkumensis* SK2	108	20	6.41
7	*Alcanivorax dieselolei* B5	151	21	4.26
8	*Alcanivorax pacificus* W11-5	121	20	4.80
9	*Alcanivorax* sp. NBRC 101098	107	21	6.78
10	*Chromohalobacter salexigens* DSM 3043	123	15	4.06
11	*Gynuella sunshinyii* YC6258	156	29	4.48
12	*Halomonas campaniensis*	133	25	3.46
13	*Hahella chejuensis* KCTC 2396	219	54	13.25
14	*Halomonas elongata* DSM 2581	122	12	2.95
15	*Halomonas huangheensis*	147	23	4.83
16	*Halomonas* sp. KO116	132	21	4.06
17	*Halomonas* sp. R57-5	141	20	3.97
18	*Marinomonas mediterranea* MMB-1	120	15	3.20
19	*Marinomonas posidonica* IVIA-Po-181	111	15	3.85
20	*Marinomonas* sp. MWYL1	122	11	2.16
21	*Oleispira antarctica* RB-8	119	15	3.40
22	*Thalassolituus oleivorans* MIL-1	117	20	5.10
23	*Thalassolituus oleivorans* R6-15	115	22	5.84


*“^∗^” means p-value < 0.01.*

S8A peptidases are the most abundant peptidase superfamily in *Kangiella* genomes. A gene cluster containing several tandem S8A superfamily peptidases (**Supplementary Figure S3**), including two orthologs (KangOF8 and 9) having multiple copies in the core genome was also identified in the *Kangiella* strains. KangOF9, which was annotated as a cold-active alkaline serine protease, was found with up to three copies per *Kangiella* genome. Interestingly, the paralogs of these proteases in KangOF9 showed overall high sequence identity, except that an additional PKD domain was observed in each of the longer ones.

## Discussion

Genomic reduction is a significant characteristic of *Kangiella* among Oceanospirillales. It was reported that both loss of entire gene families and deletion of paralogs within multigene families could contribute to genome size reduction in marine bacteria (Lerat et al., 2005), and the smaller genome size of the marine cyanobacterial genus *Prochlorococcus* resulted from gene loss compared with its closely related genus *Synechococcus* (Luo et al., 2011). Our results showed a similar mechanism of genomic reduction, including de-redundancy and genus-specific loss of orthologs, in genus *Kangiella* compared to other non-Kangiellaceae Oceanospirillales.

**FIGURE 7:**
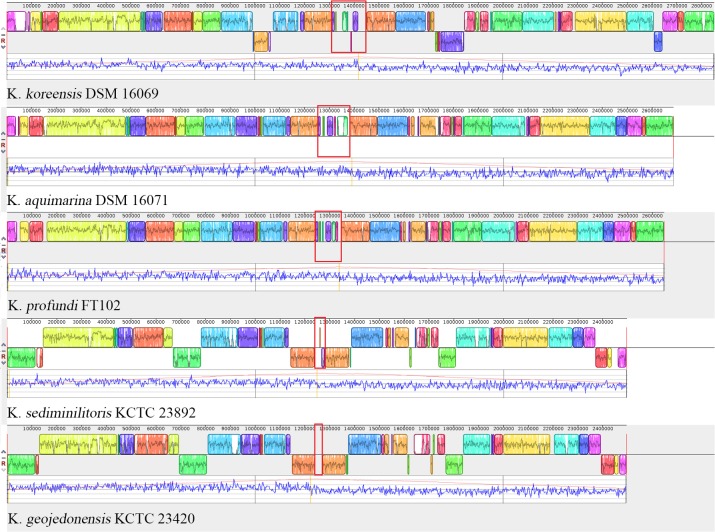
Collinearity of the *Kangiella* genomes. Highlighted sites in red show the highly variable region of each genome. Blocks in the different colors represent different locally collinear blocks (LCBs). Local GC-skew (blue) and cumulative GC-skew (red) were plotted below LCBs, and the peak position of the cumulative GC-skew was highlighted in orange.

It should be noted that in the background of global genomic reduction and de-redundancy of *Kangiella*, genomic plasticity and expansion also exist. Multigenome alignments (**Figure 7**) revealed that in the central position near the GC-skew reversion points of each *Kangiella* genome, there is an aberrant GC content genomic region (0.05–0.2 Mb) with poor colinearity. Such “hot block” for insertion and recombination was found to contain a lot of heavy metal and arsenic resistance genes in all *Kangiella* genomes, except *K. geojedonensis* KCTC 23420.

In the aspect of central metabolism, we predicted and experimentally confirmed that different *Kangiella* strains showed different defects in the capabilities of amino acid biosynthesis. For example, *K. profundi* FT102 lacks biosynthetic pathways of isoleucine, valine and phenylalanine and arginine, while *K*. *koreensis* DSM 16069 and *K*. *aquimarina* DSM 16071 only lack the first two. Interestingly, the phylogenetic tree based on 16S rRNA gene sequences showed that *K*. *aquimarina* DSM 16071 and *K. profundi* TF102 are more closely related than *K*. *koreensis* DSM 16069 (Choe et al., 2015). However, *K*. *aquimarina* DSM 16071 and *K*. *koreensis* DSM 16069 from the same sampling location showed the same capability of amino acids biosynthesis.

Furthermore, the subunits of sulfite reductase were strain-specifically missing in *K. profundi* FT102 (**Supplementary Figure S2**) as mentioned above, and we did not identify any functionally complementary gene in its genome. Actually, the sediment samples used to isolate *K. profundi* FT102 were collected from the southwest Indian InterRidge (Xu et al., 2015), which contain a large amount of sulfides and iron hydroxides (Tao et al., 2011, 2012). As long as the demand of sulfide has been satisfied from the environment, loss of those related genes seems to have no detrimental effect in *K. profundi* FT102.

In the aspect of proteolysis, our previous study confirmed the extracellular protease activities of four *Kangiella* strains (*K. profundi* FT102, *K. koreensis* DSM 16069, *K. aquimarina* DSM 16071, and *K. geojedonensis* KCTC 23420). In general, a positive correlation of the biomass and the extracellular protease activities was found when these strains were cultured in marine broth 2216 (Xu et al., 2018).

It is also worth mentioning that unlike *Aliikangiella marina* GYP-15*^T^*, which represents another genus in Kangiellaceae, and was confirmed to utilize carbohydrates (Wang et al., 2015), *Kangiella* could not grow in defined media using glucose as the sole carbon source (data not shown). Taken together, proteolysis of extracellular proteins rather than carbohydrate utilization plays an important role in the life style of *Kangiella*.

Peptidase constitute a very large and complex group of enzymes that differ in properties such as substrate specificity, active site and catalytic mechanism, pH and temperature optima, and stability profiles (Jisha et al., 2013). In the *Kangiella* strains, there are at least 43 extracellular peptidases that could be classified into 26 subfamilies. Most of them are non-redundant, indicating wide substrates specificity of peptidases in *Kangiella*. Besides, among the merely 21 gene families with redundancy in core genome of *Kangiella*, five are secreted peptidases. In detail, four (KangOF4, 8, 9 and 15) of them belong to S8 peptidase family, and KangOF19 belongs to M1 peptidase family.

The S8A family peptidases are the most abundant in *Kangiella* genomes, including the tandem gene cluster constituted with the genes of KangOF190, 9 and 8 (**Supplementary Figure S3**). S8 peptidase is the second largest family of characterized serine peptidases, and they have catalytic triad consisted of aspartate, histidine and serine (Yamagata et al., 1994), which differs from that S1, S9 and S10 serine peptidases families. S8 peptidase family could be divided into two subfamilies, i.e., subtilisin (S8A) and kexin (S8B) according to MEROPS peptidase database. The S8 peptidases are mostly secreted and non-specific peptidases, and probably involved in uptaking of nutrition, which could support the obligate proteolytic lifestyle of *Kangiella.*

Moreover, S9 family serine peptidases are also abundant in *Kangiella* (**Supplementary Table S8**). However, no redundancy of S9 family was found in the core genome of *Kangiella*. Different from S8 peptidases, most members of the S9 peptidases family show strict substrate specificity (Rawlings et al., 1991). The tendency to be streamline in the genome of *Kangiella* was evident in keeping many heterogeneous genes of S9 family members instead of gene duplication.

More interestingly, the S10 peptidase family members were only identified and are conserved in *Kanagiella* genomes compared to other genus in Oceanospirillales. Different from most other serine peptidase families (S53 being the exception), the S10 peptidases are only active at acidic pH, e.g., carboxypeptidase Y with max activity at pH 5.5 (Jung et al., 1998). As the optimal pH for the growth of *Kangiella* is 7.0–8.0, the biological function of the putative S10 peptidases in *Kangiella* is still unclear. Quantitative analysis of the expression level of S10 peptidases in *Kangiella* challenged by different pH are still needed.

In the view of evolution, it is also interesting that the duplications of the peptidase families are still obvious in the *Kangiella* genomes under the background of genomic de-redundancy. We proposed that such gene duplications are partly driven by the obligate carbon source acquisition strategy of *Kangiella*. However, *K. sediminilitoris* KCTC 23892 was an exception, which showed no abovementioned duplication of KangOF8 or KangOF9 (**Supplementary Figure S3**), as well as carriers the fewest SignalP-fused peptidases (only 69–86% of other stains) (**Table 2**). The reason might be that the duplication is not only driven by carbon acquisition but also by nitrogen acquisition. Actually, a strain-specific gene coding ammonia transporter could be only identified in *K. sediminilitoris* as mentioned above. Absorbing inorganic nitrogen from the environment might alleviate its dependency on protein-derived ammonia, thus alleviating the selective pressure that drives the multiplication of extracellular peptidase genes.

## Conclusion

In the present study, the complete genome of the novel strain *Kangiella profundi* FT102 was sequenced, and gaps in the scaffold genome of *K. aquimarina* DSM 16071 were filled using PCR. Five sequenced *Kangiella* genomes, including the abovementioned two, were utilized along with 18 non-*Kangiella* genomes in a comparative genomics approach to explore the metabolic capabilities of *Kangiella* and gain insight into genome reduction in *Kangiella*. We reported here that low genomic redundancy and *Kangiella*-lineage-specific gene loss are two key factors behind genome reduction in *Kangiella*. Furthermore, a highly enriched extracellular protein degradation system compared to that of any other non-*Kangiella* species was identified in *Kangiella*. Despite the low genome redundancy in the core genome (only 21 paralogs), five of these paralogs were signal-fused proteases. The absence of a complete pathway for carbohydrate metabolism led to the belief that proteases are not only abundant but also essential for *Kangiella* survival, demonstrating the extraordinary protein degradation capabilities of *Kangiella*.

## Author Contributions

JX and JW designed the experiments and analysis. JW and MN performed the computational analysis. YL conducted the experiments. JW, YL, and JX wrote the manuscript, in consultation with all other authors.

## Conflict of Interest Statement

The authors declare that the research was conducted in the absence of any commercial or financial relationships that could be construed as a potential conflict of interest.

## Footnotes

1 http://ftp.ncbi.nlm.nih.gov/genomes

2 http://tubic.tju.edu.cn/Ori-Finder/

3 http://weizhong-lab.ucsd.edu/metagenomic-analysis/server/cog/

4 http://www.kegg.jp/blastkoala/

5 http://genskew.csb.univie.ac.at/

## Supplementary Material

FIGURE S1 Percentage of the genes belonging to different cluster of orthologous genes (COG) categories in the orthologous gene families in pangenome of the five *Kangiella* strains. Genes belonging to the core genome, that are dispensable and that are specific to species are shown in blue, orange and gray, respectively. A: RNA processing and modification; C: Energy production and conversion; D: Cell cycle control, cell division, chromosome partitioning; E: Amino acid transport and metabolism; F: Nucleotide transport and metabolism; G: Carbohydrate transport and metabolism; H: Coenzyme transport and metabolism; I: Lipid transport and metabolism; J: Translation, ribosomal structure, and biogenesis; K: Transcription; L: Replication, recombination, and repair; M: Cell wall/membrane/envelope biogenesis; N: Cell motility; O: Posttranslational modification, protein turnover, chaperones; P: Inorganic ion transport and metabolism; Q: Secondary metabolites biosynthesis, transport, and catabolism; R: General function prediction only; S: Function unknown; T: Signal transduction mechanisms; U: Intracellular trafficking, secretion, and vesicular transport; V: Defense mechanisms; W: Extracellular structures; X: Mobilome: prophages and transposons; Z: Cytoskeleton.

FIGURE S2 Verification by PCR amplification of four *Kangiella* species, including genes involved in sulfite reduction **(A,D)** corresponding to Kkor_0726 to Kkor_0729 in the *K. koreensis* DSM 16069, genes involved in phenylalanine biosynthesis **(B,E)** corresponding to Kkor_1584 and Kkor_1585 in *K. koreensis* DSM 16069, genes involved in arginine biosynthesis (**C,F**, shown in green color) corresponding to Kkor_0543 to Kkor_0548 in *K. koreensis* DSM 16069, and genes of cytochrome c oxidase subunits (**C,G**, shown in red color) corresponding to Kkor_0534 to Kkor_0542 in *K. koreensis* DSM 16069. Ka, *K. aquimarina* DSM 16071; Kk, *K. koreensis* DSM 16069; Kg, *K. geojedonensis* KCTC 23420; Kp, *K. profundi* FT102. The numbers in **(A–C)** represent genes listed in the following: 1: cysJ, FAD-binding domain protein; 2: sulfite reductase subunit beta; 3: cysH, phosphoadenosine phosphosulfate reductase; 4: cysG, uroporphyrin-III C-methyltransferase; 5: prephenate dehydratase; 6: phospho-2-dehydro-3-deoxyheptonate aldolase; 7: cytochrome-c oxidase, cbb3-type subunit I; 8: cytochrome-c oxidase, cbb3-type subunit II; 9: CcoQ/FixQ family Cbb3-type cytochrome c oxidase assembly chaperone; 10: cytochrome-c oxidase, cbb3-type subunit III; 11: cytochrome c oxidase accessory protein CcoG; 12: nitrogen fixation protein FixH; 13: cadmium-translocating P-type ATPase; 14: cbb3-type cytochrome oxidase assembly protein CcoS; 15: sulfite exporter TauE/SafE family protein; 16: N-acetylornithine carbamoyltransferase; 17: argininosuccinate synthase; 18: acetylornithine deacetylase; 19: acetylglutamate kinase; 20: N-acetyl-gamma-glutamyl-phosphate reductase; 21: argininosuccinate lyase; 22: oxygen-independent coproporphyrinogen III.

FIGURE S3 Peptidase gene clusters (S8 FML) in the five *Kangiella* species. Arrows shown in black represent non-peptidase conserved genes. Arrows in the other colors represent the genes of different serine proteases belonging to the S8 family in the five *Kangiella* strains, including KangOF190 (orange), KangOF9 (green), KangOF8 (pink), and KangOF2260 (blue). Highlighted regions within the arrow (in red color) show the PKD domain.

TABLE S1 Number of genes belonging to each cluster of orthologous genes (COG) category in all 23 strains used in the present study.

TABLE S2 Orthologous families of the five *Kangiella* strains.

TABLE S3 Orthologous families having paralogs in the core genome of the five *Kangiella* strains.

TABLE S4 Cluster of orthologous genes (COG) distribution of the *Kangiella* pangenome.

TABLE S5 Genomic features of the 23 Oceanospirillales strains used herein.

TABLE S6 Orthologous families of the 23 Oceanospirillales strains.

TABLE S7 *Kangiella*-specific missing GOs.

TABLE S8 Number of SignalP-fused proteases identified in all 23 genomes.

MATERIAL S1 “.ab1” files and PCR primers for genomic gap filling.

MATERIAL S2 Raw gel electrophoresis maps and PCR primers corresponding to **Supplementary Figure S3**.

MATERIAL S3 Peptidase genes predicted with MEROPS batch BLAST and SignalP prediction of 23 genomes corresponding to **Supplementary Table S8**.
