# Supplementary figures and images for: Comparative Genomics Reveals Evidence of Genome Reduction and High Extracellular Protein Degradation Potential in Kangiella
Source: Front Microbiol. 2018 Jun 7;9:1224. doi: 10.3389/fmicb.2018.01224 (PMC6000758; doi:10.3389/fmicb.2018.01224)

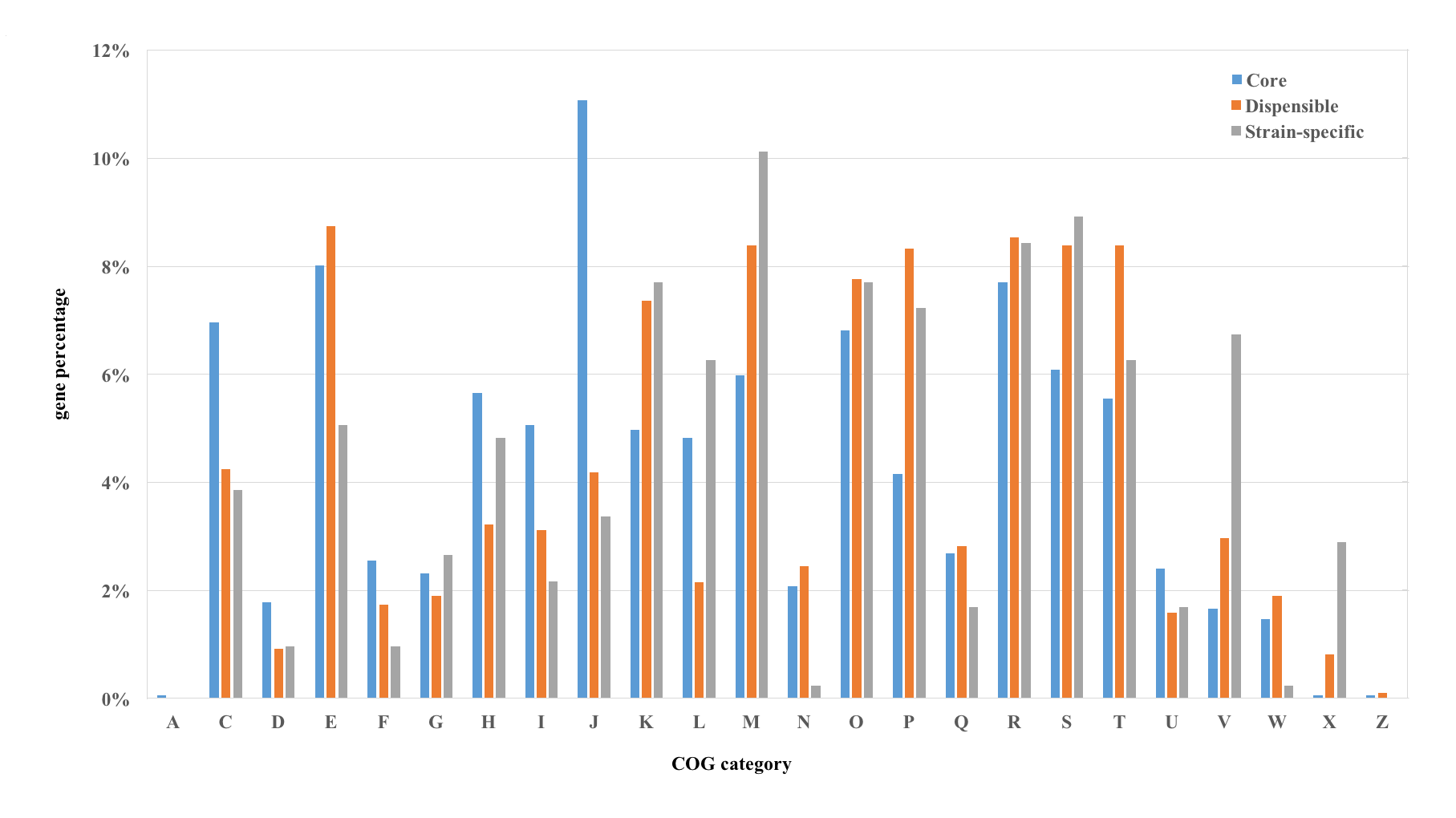

Supplement: FIGURE S1 — Percentage of the genes belonging to different cluster of orthologous genes (COG) categories in the orthologous gene families in pangenome of the five Kangiella strains. Genes belonging to the core genome, that are dispensable and that are specific to species are shown in blue, orange and gray, respectively. A: RNA processing and modification; C: Energy production and conversion; D: Cell cycle control, cell division, chromosome partitioning; E: Amino acid transport and metabolism; F: Nucleotide transport and metabolism; G: Carbohydrate transport and metabolism; H: Coenzyme transport and metabolism; I: Lipid transport and metabolism; J: Translation, ribosomal structure, and biogenesis; K: Transcription; L: Replication, recombination, and repair; M: Cell wall/membrane/envelope biogenesis; N: Cell motility; O: Posttranslational modification, protein turnover, chaperones; P: Inorganic ion transport and metabolism; Q: Secondary metabolites biosynthesis, transport, and catabolism; R: General function prediction only; S: Function unknown; T: Signal transduction mechanisms; U: Intracellular trafficking, secretion, and vesicular transport; V: Defense mechanisms; W: Extracellular structures; X: Mobilome: prophages and transposons; Z: Cytoskeleton. [file Image_1.TIF]

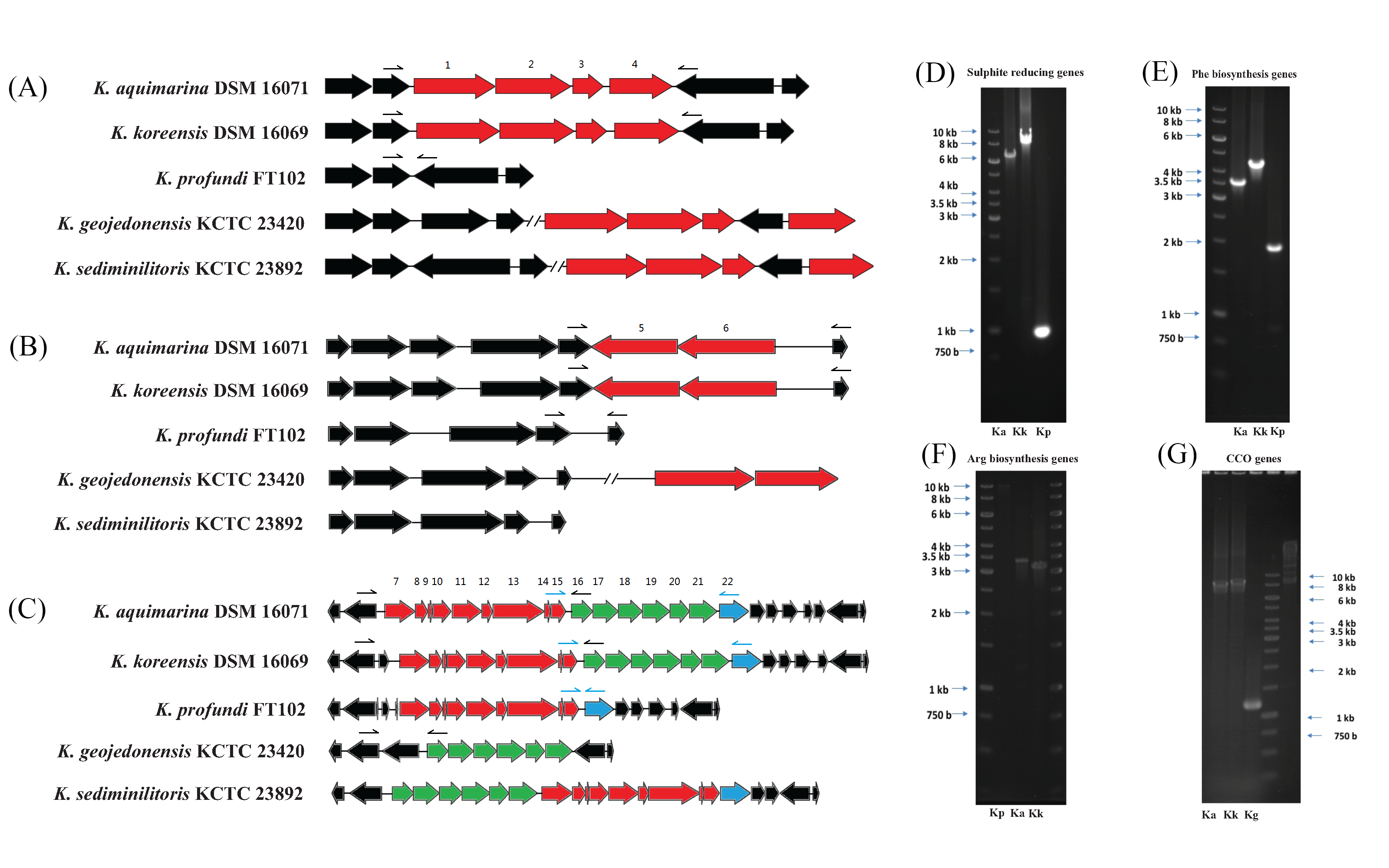

Supplement: FIGURE S2 — Verification by PCR amplification of four Kangiella species, including genes involved in sulfite reduction (A,D) corresponding to Kkor_0726 to Kkor_0729 in the K. koreensis DSM 16069, genes involved in phenylalanine biosynthesis (B,E) corresponding to Kkor_1584 and Kkor_1585 in K. koreensis DSM 16069, genes involved in arginine biosynthesis (C,F, shown in green color) corresponding to Kkor_0543 to Kkor_0548 in K. koreensis DSM 16069, and genes of cytochrome c oxidase subunits (C,G, shown in red color) corresponding to Kkor_0534 to Kkor_0542 in K. koreensis DSM 16069. Ka, K. aquimarina DSM 16071; Kk, K. koreensis DSM 16069; Kg, K. geojedonensis KCTC 23420; Kp, K. profundi FT102. The numbers in (A–C) represent genes listed in the following: 1: cysJ, FAD-binding domain protein; 2: sulfite reductase subunit beta; 3: cysH, phosphoadenosine phosphosulfate reductase; 4: cysG, uroporphyrin-III C-methyltransferase; 5: prephenate dehydratase; 6: phospho-2-dehydro-3-deoxyheptonate aldolase; 7: cytochrome-c oxidase, cbb3-type subunit I; 8: cytochrome-c oxidase, cbb3-type subunit II; 9: CcoQ/FixQ family Cbb3-type cytochrome c oxidase assembly chaperone; 10: cytochrome-c oxidase, cbb3-type subunit III; 11: cytochrome c oxidase accessory protein CcoG; 12: nitrogen fixation protein FixH; 13: cadmium-translocating P-type ATPase; 14: cbb3-type cytochrome oxidase assembly protein CcoS; 15: sulfite exporter TauE/SafE family protein; 16: N-acetylornithine carbamoyltransferase; 17: argininosuccinate synthase; 18: acetylornithine deacetylase; 19: acetylglutamate kinase; 20: N-acetyl-gamma-glutamyl-phosphate reductase; 21: argininosuccinate lyase; 22: oxygen-independent coproporphyrinogen III. [file Image_2.TIF]

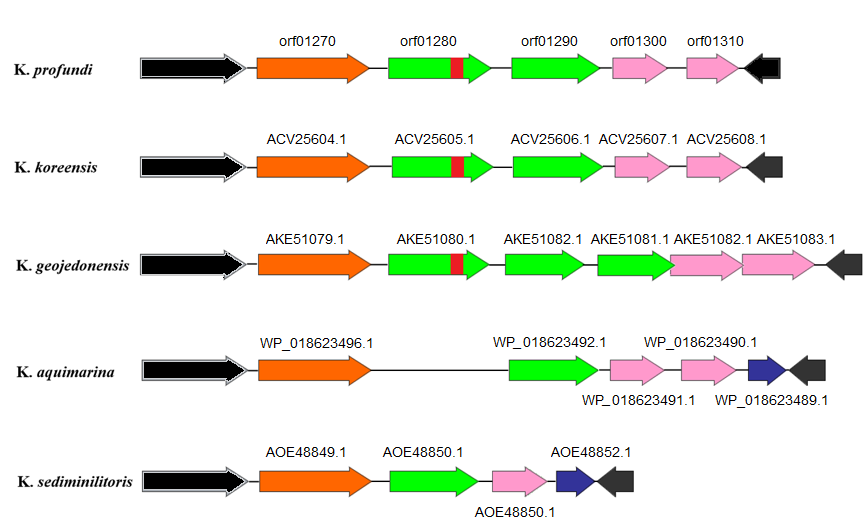

Supplement: FIGURE S3 — Peptidase gene clusters (S8 FML) in the five Kangiella species. Arrows shown in black represent non-peptidase conserved genes. Arrows in the other colors represent the genes of different serine proteases belonging to the S8 family in the five Kangiella strains, including KangOF190 (orange), KangOF9 (green), KangOF8 (pink), and KangOF2260 (blue). Highlighted regions within the arrow (in red color) show the PKD domain. [file Image_3.TIF]

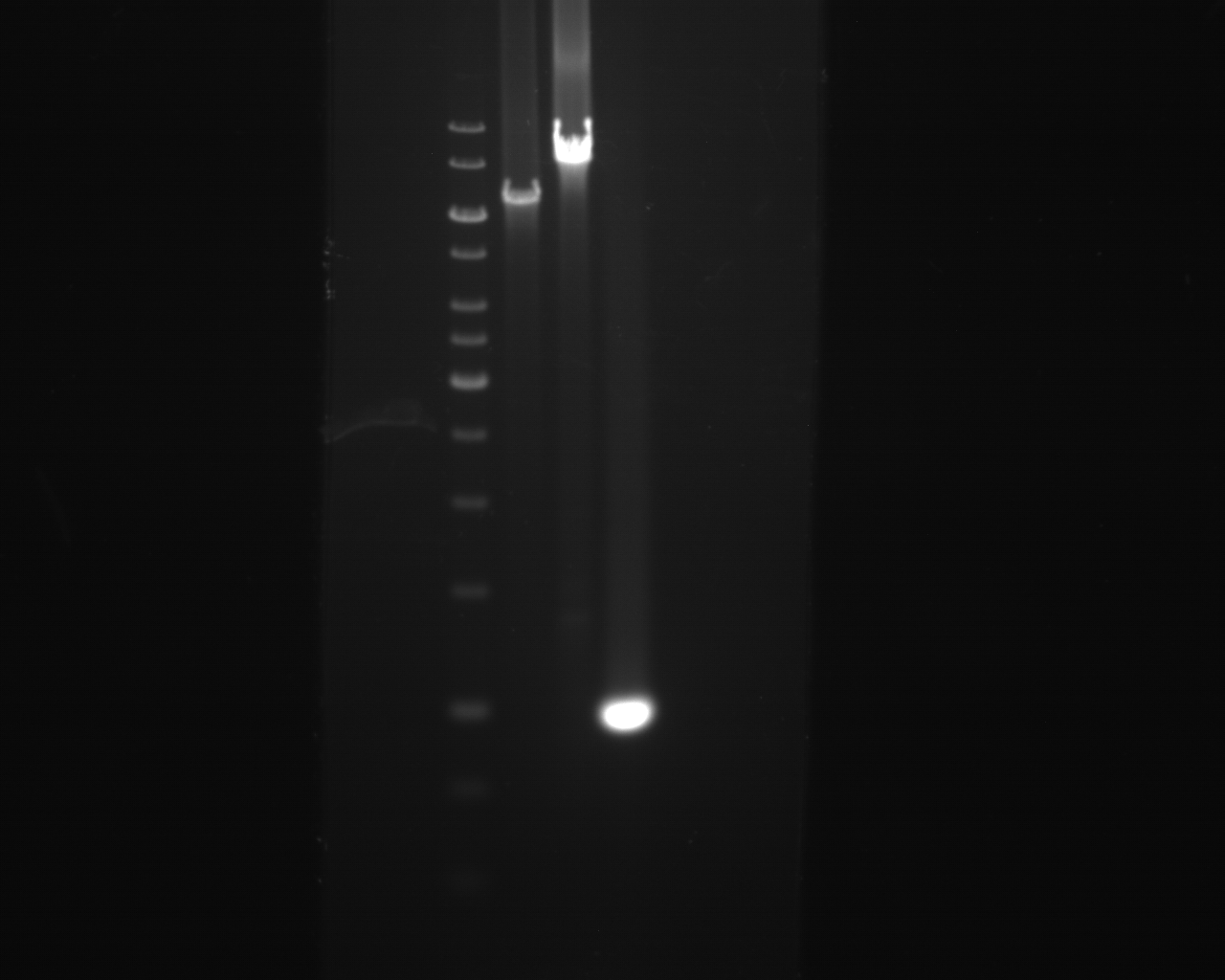

Supplement: MATERIAL S2 — Raw gel electrophoresis maps and PCR primers corresponding to Supplementary Figure S3. [file Data_Sheet_2.ZIP › Supplementary material 2/Raw_map_of_FigS2D_Sulfite.tif]

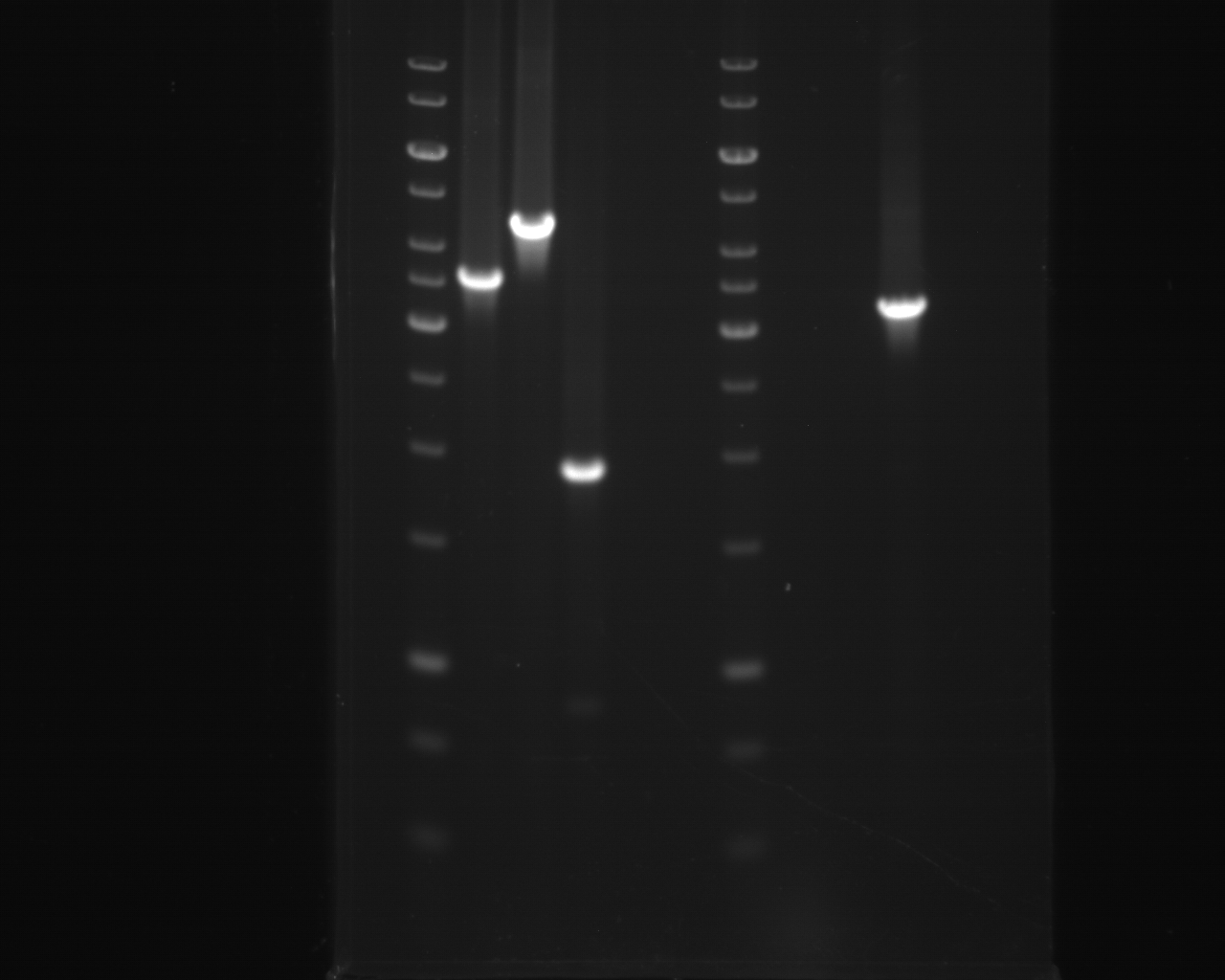

Supplement: MATERIAL S2 — Raw gel electrophoresis maps and PCR primers corresponding to Supplementary Figure S3. [file Data_Sheet_2.ZIP › Supplementary material 2/Raw_map_of_FigS2E_Phe.tif]

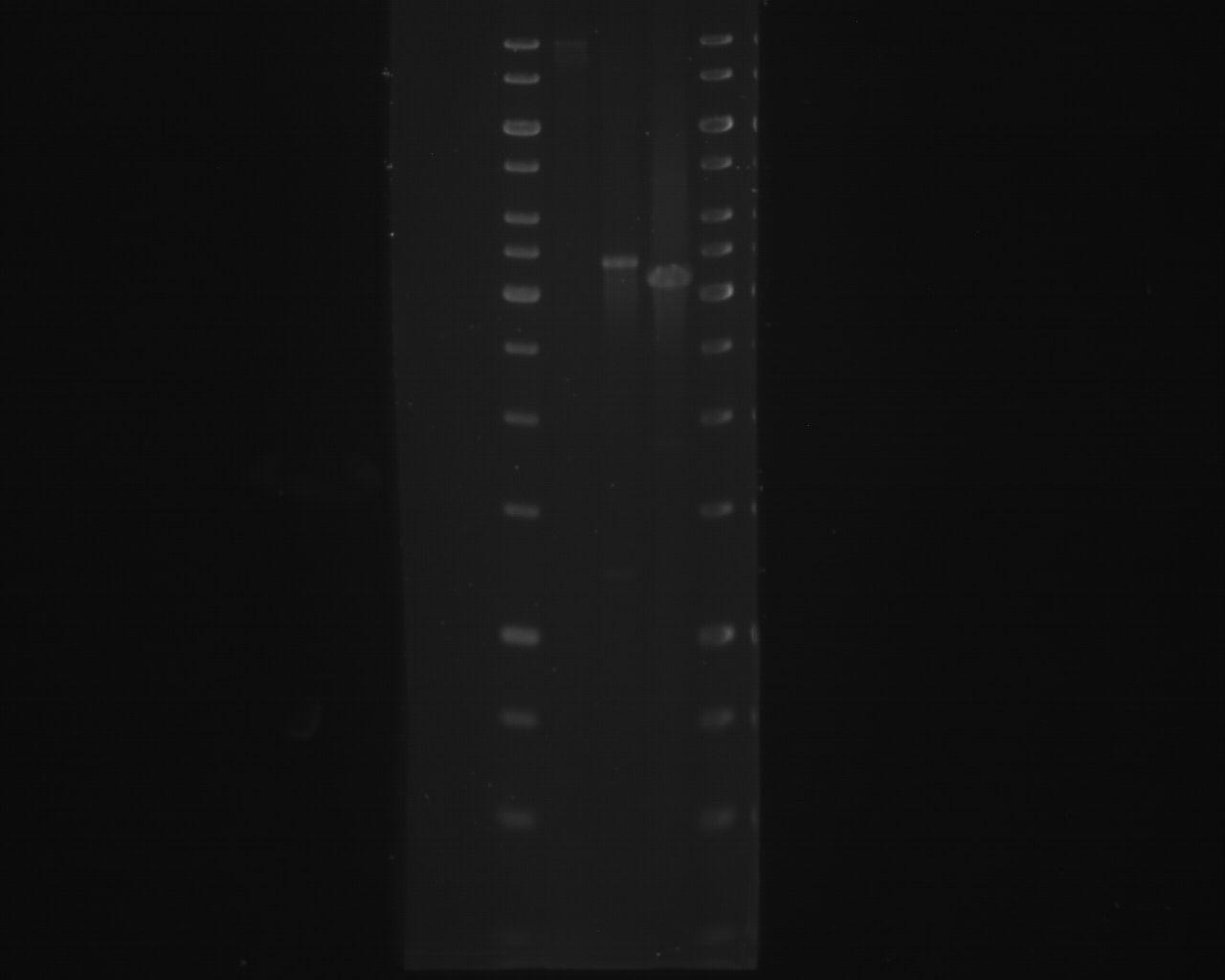

Supplement: MATERIAL S2 — Raw gel electrophoresis maps and PCR primers corresponding to Supplementary Figure S3. [file Data_Sheet_2.ZIP › Supplementary material 2/Raw_map_of_FigS2F_Arg.tif]

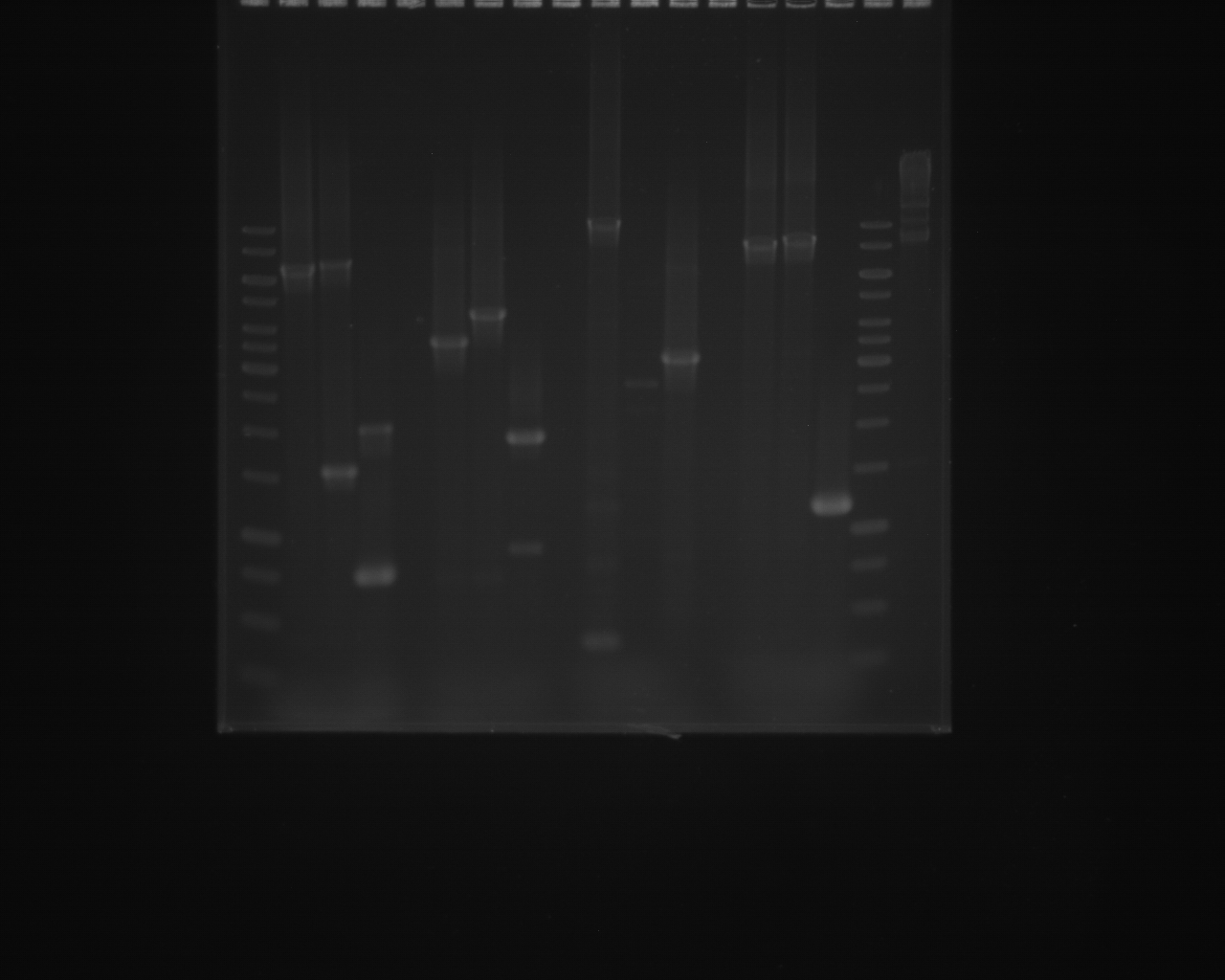

Supplement: MATERIAL S2 — Raw gel electrophoresis maps and PCR primers corresponding to Supplementary Figure S3. [file Data_Sheet_2.ZIP › Supplementary material 2/Raw_map_of_FigS2G_CCO.tif]
